# Distributed representation of pelvic floor muscles in human motor cortex

**DOI:** 10.1038/s41598-018-25705-0

**Published:** 2018-05-08

**Authors:** Moheb S. Yani, Joyce H. Wondolowski, Sandrah P. Eckel, Kornelia Kulig, Beth E. Fisher, James E. Gordon, Jason J. Kutch

**Affiliations:** 1 Division of Biokinesiology and Physical Therapy, University of Southern California, Los Angeles, CA 90033 USA; 2 Division of Biostatistics, University of Southern California, Los Angeles, CA 90033 USA

## Abstract

Human motor cortex can activate pelvic floor muscles (PFM), but the motor cortical representation of the PFM is not well characterized. PFM representation is thought to be focused in the supplementary motor area (SMA). Here we examine the degree to which PFM representation is distributed between SMA and the primary motor cortex (M1), and how this representation is utilized to activate the PFM in different coordination patterns. We show that two types of coordination patterns involving PFM can be voluntarily accessed: one activates PFM independently of synergists and a second activates PFM prior to and in proportion with synergists (in this study, the gluteus maximus muscle – GMM). Functional magnetic resonance imaging (fMRI) showed that both coordination patterns involve overlapping activation in SMA and M1, suggesting the presence of intermingled but independent neural populations that access the different patterns. Transcranial magnetic stimulation (TMS) confirmed SMA and M1 representation for the PFM. TMS also showed that, equally for SMA and M1, PFM can be activated during rest but GMM can only be activated after voluntary drive to GMM, suggesting that these populations are distinguished by activation threshold. We conclude that PFM representation is broadly distributed in SMA and M1 in humans.

## Introduction

The central nervous system has several efferent centres that cause activity in pelvic floor muscles (PFM). For example, during urine storage, low-level afferent firing produced by the bladder causes the onset of events that involve control centres at different levels in the nervous system^1–4^. First, the onset of spinal guarding reflexes may facilitate activity in the bladder base, urethra, and external urethral sphincter, while inhibiting activity in the detrusor muscle^1,2^. Second, activity in the rostral pons may result in increased activity in the external urethral sphincter^1,2^. Third, activity in the motor cortex may modulate activity in descending reticulospinal and corticospinal tracts to pelvic floor motoneurons^1–5^. The involvement of the motor cortex in controlling PFM via corticospinal projections can also be observed in other behaviours such as voluntary switching between voiding and storage, both for the bladder and for the bowel^1,2,6–8^, as well as during voluntary movements that require activation of PFM^2,9–12^. However, the contribution of the motor cortex to the control of PFM is sparsely studied; and therefore, the organization and function of the motor cortical representation of the human pelvic floor are not well understood.

Interestingly, potential dysfunction within the motor cortical representation of the pelvic floor has been described in disorders associated with pelvic pain, making this representation an important area to study. Altered cortical control of PFM has been suggested in clinical conditions, such as chronic pelvic pain and incontinence^7,13–18^, and the extent of motor cortical involvement has been suggested to predict how the patient would respond to the treatment^15,17,18^. For example, in recent resting state neuroimaging studies – when compared to healthy controls – chronic pelvic pain patients exhibited altered frequency content of the fMRI signal obtained from a pelvic-specific motor region and altered connectivity between that motor region and other brain regions^13,14^. Furthermore, in recent brain morphology studies – when compared to healthy controls – chronic pelvic pain patients displayed significant differences in gray matter volume and diffusion anisotropy in motor cortical areas consistent with control of the pelvic floor^19,20^. In incontinence patients, interaction between the periaqueductal gray (PAG) and the motor cortical representation of the pelvic floor might be altered^15^. This altered brain-bladder control can be adjusted for by learning voluntary PFM activation and urge suppression strategies^15,21^. Within this patient group, the extent of motor cortical involvement during bladder filling predicted responses to PFM training as a treatment option for urinary incontinence^15^. In stroke patients, cortical lesions can result in various bladder and bowel dysfunctions, including urinary and faecal incontinence^18^. In a case study of two patients, it has been recently suggested that stimulation of the motor cortical representation of the pelvic floor constitutes a new treatment for patients with refractory pelvic and perineal pain^17^. Therefore, characterizing the pelvic floor motor cortical representation may enable new brain-based therapies to be designed for these conditions.

A first part of this characterization is to determine whether the pelvic floor motor cortical representation is localized or distributed. A long-standing belief is that the control and representation of PFM occur in a localized region in the medial wall of the precentral gyrus toward the supplementary motor area (SMA)^1,2,9,10,12,15^. Under this hypothesis, coordination between PFM and synergistic muscles would emerge from a cortical structuring scheme involving co-activation of SMA to activate the PFM and the primary motor cortex (M1) to activate the synergist. However, this is not necessarily true. Research in animal models suggests that the role of the motor cortex in controlling voluntary movements emerges from distributed networks rather than discrete and localized representations of individual muscles^22–25^. Research in humans also points to individual muscles being represented in both SMA and M1^26^. Thus, in addition to the pelvic floor representation in SMA, the pelvic floor musculature may also have a representation in M1 that overlaps with the representation of muscles with which the PFM synergistically co-active. We have previously reported indirect evidence of this overlapping representation between pelvic floor and lower limb muscles in M1^9,11^. Therefore, in the present study we used a more refined paradigm to map the PFM motor cortical representation in which we carefully scaled cortical activity to different levels using voluntary muscle activation, and determined the response in SMA and M1 using both functional magnetic resonance imaging (fMRI) and transcranial magnetic stimulation (TMS).

## Results

### Summary

Electromyography (EMG) results showed that two types of coordination patterns involving pelvic floor muscles (PFM) can be voluntarily accessed: an “isolated-pelvic” coordination pattern that activates PFM independently of synergists, in this study, the gluteus maximus muscle – GMM; and a “gluteal-pelvic” coordination pattern that activates PFM prior to and in proportion with the synergistic GMM. Functional magnetic resonance imaging (fMRI) results showed that both coordination patterns involve overlapping activation in SMA and M1, suggesting the presence of intermingled but independent neural populations that access the different coordination patterns. TMS results confirmed a representation of the PFM distributed in both SMA and M1. Similarly, for each motor region (SMA and M1), TMS results showed that PFM potentials can be evoked during rest but GMM potentials can only be evoked after voluntary pre-activation of GMM, suggesting that these intermingled populations may be distinguished by activation threshold.

### EMG characterization of coordination patterns

**Figure 1:**
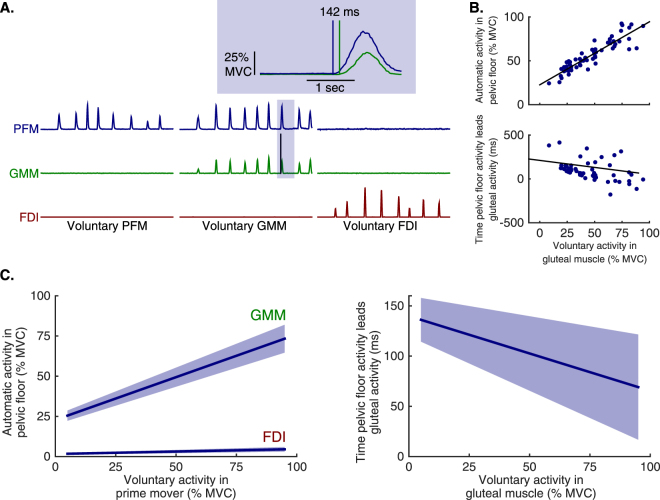
Electromyographic (EMG) evidence of two muscle coordination patterns involving human pelvic floor muscles (PFM) that the brain can voluntarily access. (**A**) Example EMG recordings from the PFM (blue), gluteus maximus muscle (GMM, green), and the first dorsal interosseous muscle (FDI, red) in a single participant during repeated and separate voluntary activations of the PFM, GMM, and FDI. Voluntary activation of the PFM elicits the isolated-pelvic coordination pattern which does not have co-activation of the GMM or FDI. Voluntary activation of the GMM activates the gluteal-pelvic coordination pattern which co-activates the GMM and PFM. The inset shows one example voluntary GMM activation, demonstrating that coordinated PFM activity occurred in advance of GMM activity. (**B**) Example of EMG amplitude and latency in a single participant as voluntary GMM activation is increased. Since all participants performed repeated and scaled muscle contractions, each participant provided EMG data that were used in the group analyses. (**C**) *left*. Population average (solid line, ± SEM) PFM activation – separately during voluntary GMM activation and voluntary FDI activation – from a linear mixed model with participant-level random intercepts and slopes. Increases in PFM activation associated with increases in voluntary GMM activation were significantly larger than those associated with increases in voluntary FDI activation (p < 0.005). (**C**) *right*. Population average (solid line, ± SEM) PFM activation latency relative to the onset of the GMM – during voluntary GMM activation – from a linear mixed model with participant-level random intercepts and slopes. PFM activation led GMM activation across the range of voluntary GMM activation, with slightly shorter latencies for higher levels of voluntary GMM activation (p = 0.03).

Our EMG recordings confirmed the existence of the isolated-pelvic and gluteal-pelvic coordination patterns we have described previously^9^. Cuing participants to voluntarily contract their PFM did not generate any associated activity in either the GMM or the first dorsal interosseous muscle (FDI, Fig. 1A). Cuing participants to voluntarily contract their GMM induced coupled activity in the PFM, and this activity led GMM activity by over 100 ms (Fig. 1A – inset). Voluntary activation of the FDI activated neither the PFM nor the GMM (Fig. 1A). In contrast to our prior work^9^, here we also varied the intensity of the primary muscle activation to look for evidence of coupled and coordinated neural drive to the PFM. We found that increasing GMM voluntary activation was associated with an increase in PFM co-activation and a decrease in the onset latency measure that was the difference between PFM onset and GMM onset, both in an example participant (Fig. 1B) and in group data (Fig. 1C, Table 1). Linear mixed effects regression models applied to the population of participants verified the following (see detailed statistical results in Table 1).

**Table 1:** Electromyographic (EMG) results during scaled voluntary muscle activation.

**A. PFM co-activation magnitude during GMM scaled voluntary activation and during FDI scaled voluntary activation**		
	**PFM during GMM** Fig. 1C, left	**PFM during FDI** Fig. 1C, left
**Population Mean Slope**	0.5320 [%-PFM-MVC per %-GMM-MVC]	0.0306 [%-PFM-MVC per %-FDI-MVC]
standard error (SE)	0.0608	0.0100
p-value	p < 0.0001	p = 0.0039
95% confidence interval	0.4091, 0.6548	0.0100, 0.0508
**Population Mean Intercept** [%-PFM-MVC]	22.7975	1.5960
standard error (SE)	2.8943	0.5070
p-value	p < 0.0001	p = 0.0031
95% confidence interval	16.9524, 28.6427	0.5714, 2.6206
**GMM co-activation magnitude during PFM scaled voluntary activation**		
	**GMM during PFM**	
**Population Mean Slope** [%-GMM-MVC per %-PFM-MVC]	0.0251	
standard error (SE)	0.0049	
p-value	p < 0.0001	
95% confidence interval	0.0151, 0.0351	
**Population Mean Intercept** [%-GMM-MVC]	0.4364	
standard error (SE)	0.1589	
p-value	p = 0.0089	
95% confidence interval	0.1156, 0.7573	
**B. PFM co-activation latency during GMM scaled voluntary activation**		
	**PFM during GMM** Fig. 1C, right	
**Population Mean Slope** [ms per %-GMM-MVC]	−0.7444	
standard error (SE)	0.3404	
p-value	p = 0.0345	
95% confidence interval	−0.05701, −1.4318	
**Population Mean Intercept** [ms: milliseconds, PFM leading GMM]	139.85	
standard error (SE)	20.0510	
p-value	p < 0.0001	
95% confidence interval	180.35, 99.3596	

PFM/FDI relation may not be meaningful since the increase in PFM co-activation magnitude is greater during GMM voluntary activation compared to voluntary FDI activation. Fig. 1C, left: estimated slope difference = 0.5066, SE = 0.1621, p = 0.0033, 95%CI (0.1790, 0.8343). PFM co-activation magnitude is greater during low GMM voluntary activation compared to low voluntary FDI activation. Fig. 1C, left: estimated intercept difference = 21.1847 [%-PFM-MVC], SE = 2.5868, p < 0.0001, 95%CI (15.9564, 26.4129).

The increase in PFM co-activation magnitude during GMM voluntary activation is greater than the increase in GMM co-activation magnitude during PFM voluntary activation. Estimated slope difference = 0.4340, SE = 0.0115, p < 0.0001), 95%CI (0.4115, 0.4565). PFM co-activation magnitude during low GMM voluntary activation is greater than GMM co-activation magnitude during low PFM voluntary activation (estimated intercept difference = 24.0397, %-MVC, SE = 3.2886, p < 0.0001, 95%CI (17.3982, 30.6812).

Positive co-activation latency means that PFM co-activation happened before the associated GMM voluntary activation.

Statistics are based on linear mixed effects regression models; see METHODS section.

PFM (pelvic floor muscles); GMM (gluteus maximus muscle); FDI (first dorsal interosseous muscle).

First, PFM co-activation magnitude increases with increasing GMM voluntary activation magnitude (Fig. 1C, left; Table 1A). Second, PFM co-activation magnitude does slightly increase with increasing FDI voluntary activation magnitude (Fig. 1C, left; Table 1A). However, we do not consider PFM/FDI relation to be meaningful since the increase in PFM co-activation magnitude is greater during GMM voluntary activation compared to voluntary FDI activation (Fig. 1C, left; Table 1A), and, PFM co-activation magnitude is greater during low GMM voluntary activation compared to low voluntary FDI activation (Fig. 1C, left; Table 1A). Third, GMM co-activation magnitude does slightly increase with increasing PFM voluntary activation magnitude (Table 1A). However, the increase in PFM co-activation magnitude during GMM voluntary activation is greater than the increase in GMM co-activation magnitude during PFM voluntary activation (Table 1A), and, PFM co-activation magnitude during low GMM voluntary activation is greater than GMM co-activation magnitude during low PFM voluntary activation (Table 1A). Fourth, increasing GMM voluntary activation decreases the timing of PFM co-activation relative to the onset of GMM voluntary activation (Fig. 1C, right; Table 1B). As expected, population mean intercept was significant, as PFM led the GMM by ~140 milliseconds (Fig. 1C, right; Table 1B).

To summarize, human PFM can be activated independently of GMM across a wide range of activation intensities, but PFM co-activate in proportion to and in advance of GMM during voluntary GMM activation. PFM activity leads GMM activity by a shorter duration as voluntary GMM activity becomes more intense, but PFM activity does not become synchronous with or trail GMM activity for any contraction intensity. The asymmetry in coupling between the PFM and GMM points toward independent neural populations in the motor cortex that are used to control the isolated-pelvic and gluteal-pelvic coordination patterns, but does not localize these populations as we begin to in the next section.

### fMRI localization of motor cortical control centres

**Figure 2:**
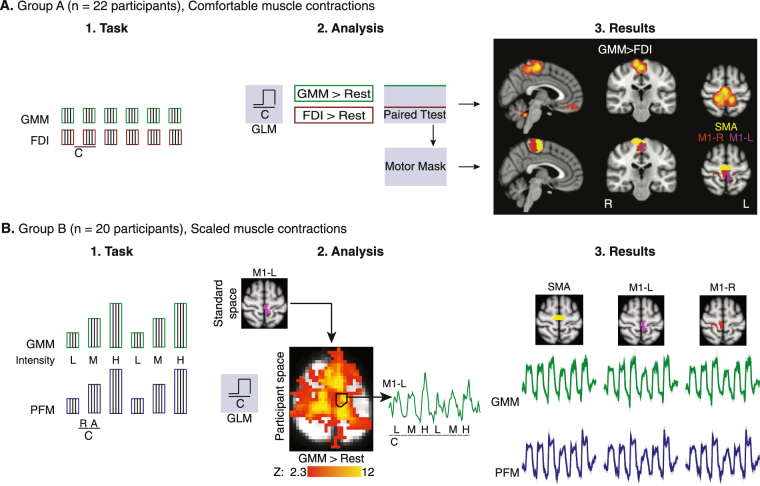
Functional magnetic resonance imaging (fMRI) evidence of non-localized control of human pelvic floor muscles (PFM). (**A**-**1**) fMRI data were collected while a first group of participants (Group A) voluntarily activated the first dorsal interosseous muscle (FDI) and the gluteus maximus muscle (GMM) in blocks at a comfortable level. (**A-2,3**) Contrast of voluntary GMM activation greater than voluntary FDI activation produced significant brain activation in distinct brain areas (Z > 2.3, p < 0.05, cluster corrected), including three distinct ROIs in the motor cortex: supplementary motor cortex (SMA) and bilateral primary motor cortex (M1-L and M1-R). (**B-1**) fMRI data were collected while a different set of participants (Group B) voluntarily activated the GMM and PFM in blocks to three distinct levels: low (L), medium (M), and high (H). (**B-2**) The motor cortical ROIs (SMA, M1-L, M1-R) were projected into the native space to identify voxels in these ROI that were active (Z > 2.3, p < 0.05, cluster corrected) in the contrast of muscle activity compared to rest, regardless of contraction level. For each participant and ROI, BOLD signals extracted from the identified voxels were averaged. (**B-3**) Activation significantly increased in all ROIs during increased voluntary GMM activation (gluteal-pelvic coordination pattern) and during increased voluntary PFM activation (isolated-pelvic coordination pattern). Specifically, activity significantly increased in SMA during increased voluntary GMM (p < 0.0001), in M1-L during increased voluntary GMM (p < 0.0001), in M1-R during increased voluntary GMM (p < 0.0001), in SMA during increased voluntary PFM (p < 0.0001), in M1-L during increased voluntary PFM (p < 0.0001), and in M1-R during increased voluntary PFM (p < 0.0001).

We used two independent groups of participants in our fMRI analysis to enhance the validity of our findings. First, using fMRI data collected while a first group of participants (Group A) voluntarily activated FDI and GMM in blocks at a comfortable level, we observed significant brain activation in the contrast (GMM > FDI, Fig. 2A) in distinct brain areas (Z > 2.3, p < 0.05, cluster corrected), including three ROIs in the motor cortex: SMA, left M1 (M1-L) and right M1 (M1-R). In each ROI, all voxels achieved statistical significance in the contrast of voluntary GMM activation greater than voluntary FDI activation, and had at least 25% probability of belonging to a certain Brodmann area (BA: BA6 for SMA, BA4L for M1-L, BA4R for M1-R) based on the Jülich Histological Atlas. M1-L and M-1R were similar in size (453 voxels in M1-L and 441 voxels in M1-R). Second, using fMRI data collected while a different set of participants (Group B) voluntarily activated the GMM and PFM in blocks to three distinct levels: low (L), medium (M), and high (H), we observed similar changes in brain activity (as measured by BOLD signal strength) in the identified ROIs (SMA, M1-L, M1-R) during GMM scaled voluntary activation as well as during PFM scaled voluntary activation (Fig. 2B). PFM scaled voluntary activation in the primary motor cortex was symmetric: at the individual level, we observed similar number of active voxels in M1-L and M1-R (paired t-test: p = 0.1244). Activation in each ROI did not depend strongly on the gender (unpaired t-test: M1-L, p = 0.0591; M1-R, p = 0.0612).

We have hypothesized that scaling GMM voluntary activation magnitude, i.e., scaling the voluntary drive to elicit the gluteal-pelvic coordination pattern, would be associated with scaling of brain activity in motor regions related to the control of the GMM, and possibly to the control of the PFM – when assuming localized representation of the PFM in SMA and cortical structuring of the gluteal-pelvic coordination pattern. Our ANCOVA analysis (see METHODS) of the averaged BOLD signals, extracted from each ROI in each participant in Group B, showed scaling in BOLD signal strength in accordance to the activation level of GMM in the three identified motor ROIs. Specifically, on the population level, BOLD signal strength significantly increased in SMA, M1-L, and M1-R during increased voluntary GMM activation (Table 2A).

**Table 2:** Functional MRI (fMRI) BOLD scaling during scaled voluntary muscle activation.

**A. ANCOVA results in Group B, during scaled GMM voluntary activation** Testing for scaling in averaged BOLD signal extracted from three pre-defined ROIs from Group A			
**β**_**1**_ **comparing BOLD strength during L to strength during H**	**SMA**	**M1-L**	**M1-R**
**Estimated Mean Difference (L-H)**	−79.8394	−61.0844	−71.5869
standard error (SE)	15.1499	8.8635	14.4091
p-value	p < 0.0001	p < 0.0001	p < 0.0001
95% confidence interval	−109.91, −49.711	−78.6760, −43.4929	−100.1800, −42.9889
**β**_**2**_ **comparing BOLD strength during M to strength during H**			
**Estimated Mean Difference (M-H)**	−49.8340	−42.1037	−49.3905
standard error (SE)	14.3108	8.1451	13.3617
p-value	p = 0.0007	p < 0.0001	p = 0.0004
95% confidence interval	−78.2371, −21.4310	−58.2695, −25.9378	−75.9097, −22.8713
**B. ANCOVA results in Group B, during scaled PFM voluntary activation** Testing for scaling in averaged BOLD signal extracted from three pre-defined ROIs from Group A			
**β**_**1**_ **comparing BOLD strength during L to strength during H**	**SMA**	**M1-L**	**M1-R**
**Estimated Mean Difference (L-H)**	−29.4630	−38.1871	−39.1023
standard error (SE)	4.8281	5.2601	5.8865
p-value	p < 0.0001	p < 0.0001	p < 0.0001
95% confidence interval	−39.0455, −19.8805	−48.6270, −27.7473	−50.7854, −27.4192
**β**_**2**_ **comparing BOLD strength during M to strength during H**			
**Estimated Mean Difference (M-H)**	−28.9518	−34.9262	−33.9300
standard error (SE)	4.7456	5.2520	5.9434
p-value	p < 0.0001	p < 0.0001	p < 0.0001
95% confidence interval	−38.3705, −19.5330	−45.3499, −24.5024	−45.7259, −22.1340

Scaling in BOLD signal strength during three distinct levels of voluntary activation, low (L), medium (M), and high (H), is investigated using an ANCOVA model. See MEHODS section. A_ij_ = Intercept + β_1_ * L + β_2_ * M + 0 * (H) + β_3_ * R_ij_ + U_i_ + E_ij_ A_ij_ is the 75 percentile of the pre-assumed Active period, while R_ij_ is the 50 percentile of the preceding pre-assumed Rest period. L is identifier of low activation, M is identifier of medium activation, and H is identifier of high activation.

Each participant is assumed to have a unique intercept [Intercept + Ui].

SMA (supplementary motor area); M1-L (primary motor cortex, left hemisphere); M1-R (primary motor cortex, right hemisphere).

Based on previous studies^9–12,27,28^, we hypothesized that scaling BOLD signal strength in SMA would be to activate and scale PFM activity and that scaling BOLD signal strength in M1-L and M1-R would be to activate and scale GMM activity. However, the following fMRI results during scaling of the isolated-pelvic coordination pattern did not validate this initial hypothesis of localized control and representation of the PFM in SMA. Our ANCOVA analysis of BOLD signal strength showed scaling in BOLD signal strength in accordance to the activation level of PFM not only in SMA ROI but also bilaterally in the primary motor cortex in M1-L and M1-R ROIs. Specifically, on the population level, BOLD signal strength significantly increased in SMA, M1-L, and M1-R during increased voluntary PFM activation (Table 2B). In contrast to previous studies, we can conclude that cortical control of human PFM may extend to motor regions lateral to the medial wall of the motor cortex.

To summarize, the above fMRI results could not validate our initial hypotheses of localized control and representation of the PFM in SMA, and that during voluntary and scaled GMM activation, scaling of brain activity in SMA would be to activate and scale PFM activity and that scaling of brain activity in M1-L and M1-R would act solely to activate and scale GMM activity. Therefore, the TMS study was particularly required to determine whether the cortical representation of the PFM is localized in SMA or distributed in the motor cortex.

### TMS evidence of non-localized control of human PFM

**Figure 3:**
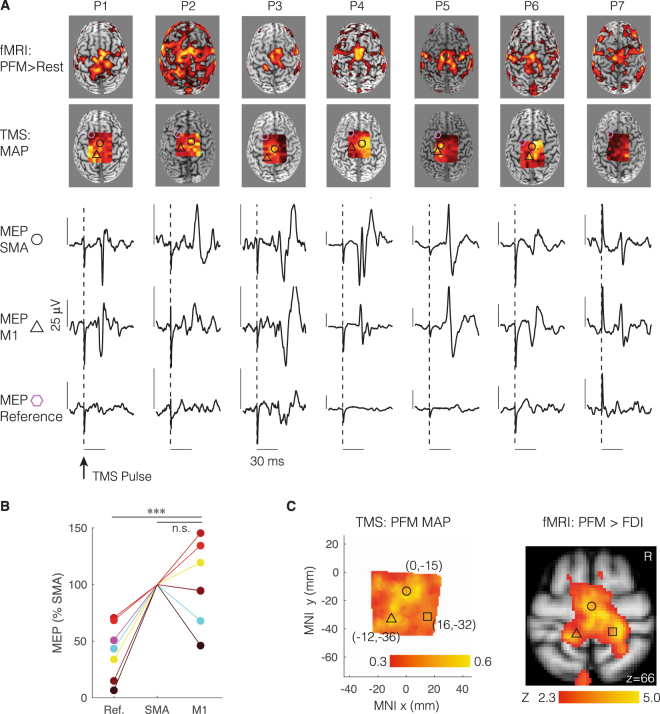
Transcranial magnetic stimulation (TMS) evidence of distributed motor cortical representation of human pelvic floor muscles (PFM). (**A**) Individual data from 7 participants who had both functional magnetic resonance (fMRI) and TMS testing. First row of images shows fMRI activation maps for the contrast PFM greater than rest (Z > 2.3, p < 0.05, corrected). Notice the distributed activation extending posterior and lateral to medial supplementary motor area (SMA), consistent with primary motor cortex (M1). Second row of images shows TMS maps of motor evoked potentials (MEPs) generated in the PFM during stimulation at rest. Notice again the MEPs did not appear confined to the medial wall and SMA. Shapes show locations chosen by the experimenter during the TMS session for further analysis: significant MEP and overlying SMA (circles), significant MEP and overlying left M1 (triangles), and a non-significant MEP reference site (hexagon). Subsequent rows show MEPs (averaged across 3 stimuli) evoked in the PFM from these locations in these participants. Notice that MEPs appeared in the PFM 21 milliseconds (ms) after the TMS pulse, consistent with direct (though not necessarily monosynaptic) connections, and that MEPs evoked from M1 were not generally smaller – and in some cases larger – than MEPs evoked from SMA. (**B**) Statistical analysis of MEP peak-to-peak size (normalized to SMA) revealed that PFM MEPs induced by stimulating M1 were significantly larger than PFM MEPs induced by stimulating the reference (p < 0.001), but were not significantly different from PFM MEPs induced by stimulating SMA (p > 0.05). (**C**) The TMS map for the PFM (averaged across participants) broadly mirrored the fMRI group activation map just under the scalp (z = 66 mm, MNI coordinates) for the contrast of PFM greater than first dorsal interosseous (FDI). Example coordinates chosen according to the Jülich anatomical atlas consistent with SMA (circle), left M1 (triangle) and right M1 (square) are shown for reference.

Confirming our fMRI findings, we found that hotspots for the PFM were not confined over a single cortical region (Fig. 3). Inspecting individual fMRI and TMS data from 7 participants who had both procedures revealed (1) distributed activation in the contrast PFM greater than rest extending posterior and lateral to medial SMA (consistent with M1), and (2) PFM motor evoked potentials (MEPs) did not appear confined to the medial wall and SMA (Fig. 3A). MEP size (normalized to SMA) revealed that PFM MEPs induced by stimulating M1 were significantly larger than PFM MEPs induced by stimulating a non-motor reference region (Fig. 3B; p < 0.001, paired t-test), but were not significantly different from PFM MEPs induced by stimulating SMA (Fig. 3B; p = 0.99, paired t-test). The TMS map of the PFM, averaged across participants, broadly mirrored the fMRI activation map of the PFM (Fig. 3C).

**Figure 4:**
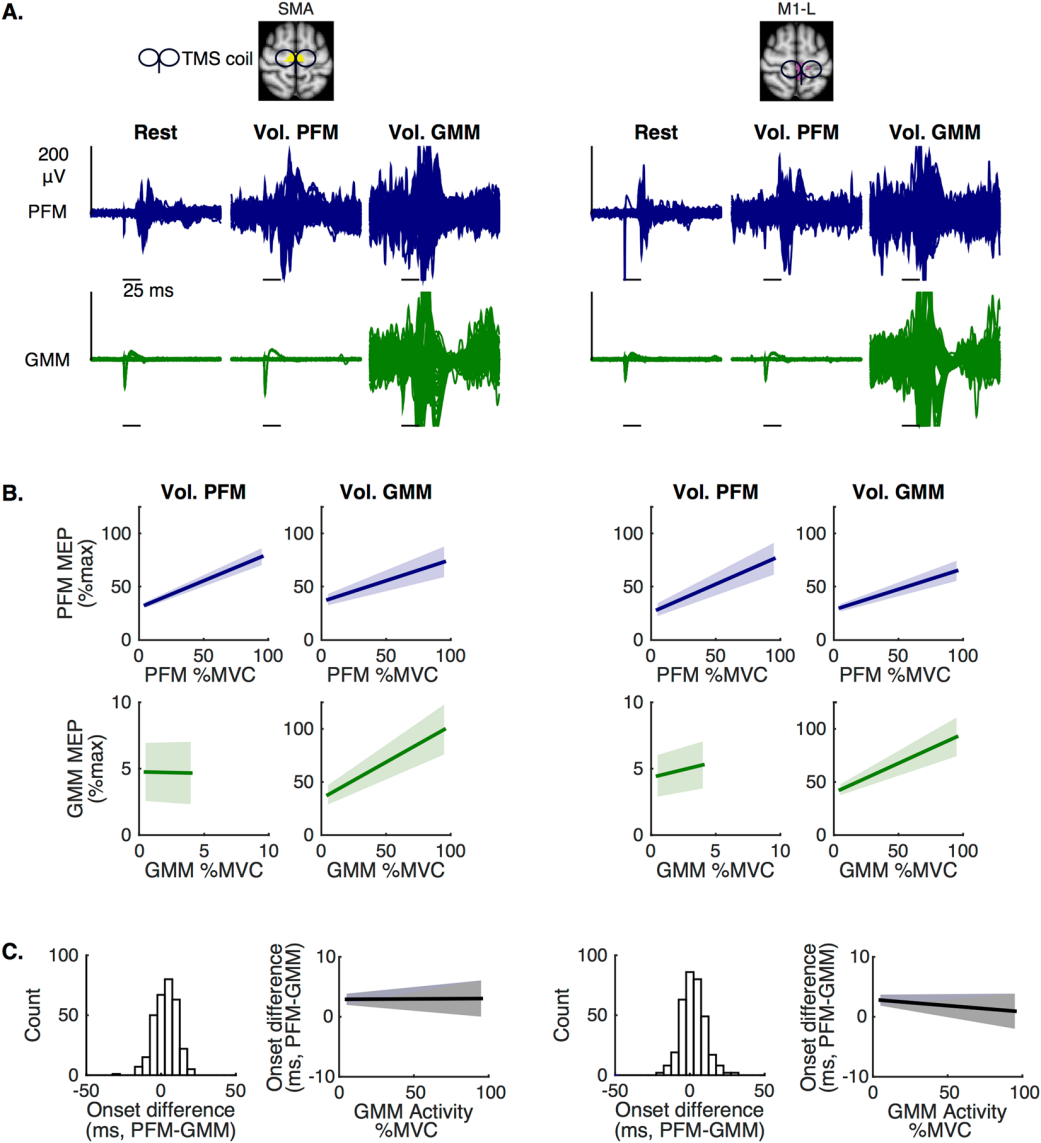
Transcranial magnetic stimulation (TMS) evidence of increasing neural activation in human supplementary motor area (SMA) and primary motor cortex (M1) during increased voluntary drive. (**A**) Pelvic floor muscles (PFM) hotspots in SMA and M1-L were separately stimulated to test whether MEPs can be elicited in the PFM (top row) and the gluteus maximus muscle (GMM, bottom row) at rest (first column), during PFM voluntary activation (second column), and during GMM voluntary activation (third column). Plotted MEPs were limited to contraction levels ≤ 25% MVC. PFM MEPs were observed at rest and during PFM and GMM voluntary activations. However, no GMM MEPs were observed at rest or during PFM voluntary activation. GMM MEPs were only observed during GMM voluntary activation. (**B**) Population average (solid line, ± SEM) MEPs peak-to-peak values (amplitudes) from a linear mixed model with participant-level random intercepts and slopes. In both motor regions, increased PFM activation resulted in significantly increased amplitudes of PFM MEPs (PFM-SMA: p = 0.0001, PFM-M1-L: p = 0.0017). However, increasing PFM activation did not result in increasing either GMM activation level or GMM MEPs amplitudes. In both motor regions, increasing GMM activation resulted in modulating PFM activation level, as well as, in modulating amplitudes of PFM and GMM MEPs (PFM-SMA: p = 0.0076, PFM-M1-L: p = 0.0011, GMM-SMA: p = 0.0052, GMM-M1-L: p = 0.009). (**C**) Distribution of the difference between onset latency of PFM MEPs and onset latency of corresponding GMM MEPs during GMM voluntary activation. Using linear mixed model with participant-level random intercepts and slopes showed that increasing GMM activation was not associated with changes in the difference (latency of PFM MEPs - latency of GMM MEPs, SMA: p = 0.9539, M1-L: p = 0.3954) that remained about +3 ms on average and was significantly greater than zero (SMA: p = 0.0121, M1-L: p = 0.0114).

More detailed stimulation over the SMA hotspot for the PFM (Fig. 4, left) or the M1 hotspot for the PFM (Fig. 4, right) revealed very similar findings. First, we note that it was possible to induce MEPs in the PFM at rest, but not possible to induce MEPs in the GMM at rest or during PFM voluntary activation; however, as previously observed^28^, it was possible to induce MEPs in the GMM during GMM voluntary activation (Fig. 4A). This result immediately suggests that there are two neural populations in the motor cortex related to the activation of these muscles, and that these populations are distinguished by baseline excitability.

Additionally, stimulating either PFM hotspots during different levels of PFM voluntary pre-activation – then during different levels of GMM voluntary pre-activation – resulted in modulating MEPs induced in the PFM, and in the PFM and the GMM, respectively (Fig. 4B, Table 3A,B). Changes in MEP magnitude with changes in voluntary activation were significant. First, in activating the isolated-pelvic coordination pattern, we found that scaling PFM voluntary activation was associated with modulation in peak-to-peak values of MEPs induced in the PFM (Fig. 4B: columns 1 & 3, 1^st^ row; Table 3A), but was not associated with induction of MEPs in the GMM (Fig. 4B: columns 1 & 3, 2^nd^ row; Table 3B). Second, in activating the gluteal-pelvic coordination pattern, we found that scaling GMM voluntary activation was associated with modulation in peak-to-peak values of MEPs induced in the PFM (Fig. 4B: columns 2 & 4, 1^st^ row; Table 3A) and in the GMM (Fig. 4B: columns 2 & 4, 2^nd^ row; Table 3B).

**Table 3:** Motor evoked potentials (MEPs) during scaled voluntary muscle activation.

**A. Response in muscle group of interest (PFM)**	**SMA**		**M1-L**	
**PFM MEP (% max)**	**Vol. PFM**	**Vol. GMM**	**Vol. PFM**	**Vol. GMM**
**Population Mean Slope** [%-PFM-MAX-MEP per %-PFM-MVC]	0.5048	0.3951	0.5324	0.3864
standard error (SE)	0.0574	0.1003	0.0992	0.0662
p-value	p = 0.0001	p = 0.0076	p = 0.0017	p = 0.0011
95% confidence interval	0.3642, 0.6453	0.1497, 0.6405	0.2897, 0.7751	0.2244 to 0.5484
**Population Mean Intercept** [%-PFM-MAX-MEP]	30.1635	35.7143	25.5859	28.0732
standard error (SE)	2.4474	4.8129	5.5339	3.1934
p-value	p < 0.0001	p = 0.0003	p = 0.0036	p = 0.0001
95% confidence interval	24.1750, 36.1521	23.9377, 47.4910	12.0451, 39.1268	20.2591, 35.8873
**B. Response in muscle group of interest (GMM)**	**SMA**		**M1-L**	
**GMM MEP (% max)**	**Vol. PFM**	**Vol. GMM**	**Vol. PFM**	**Vol. GMM**
**Population Mean Slope** [%-GMM-MAX-MEP per %-GMM-MVC]	−0.0230	0.6835	0.2369	0.5534
standard error (SE)	0.0484	0.1599	0.0572	0.1458
p-value	p = 0.6506	p = 0.0052	p = 0.0061	p = 0.0090
95% confidence interval	−0.1413, 0.0953	0.2924, 1.0747	0.0970, 0.3768	0.1966, 0.9101
**Population Mean Intercept** [%-GMM-MAX-MEP]	4.7665	34.5084	4.3409	39.9516
standard error (SE)	2.1595	8.2745	1.5374	4.3683
p-value	p = 0.0694	p = 0.0059	p = 0.0302	p < 0.0001
95% confidence interval	−0.5175, 10.0505	14.2615, 54.7553	0.5791, 8.1028	29.2628, 50.6405
**C. Onset of MEP during GMM scaled voluntary activation**	**SMA**		**M1-L**	
	**MEP in PFM**	**MEP in GMM**	**MEP in PFM**	**MEP in GMM**
**Population Mean Slope** [ms per %-GMM-MVC]	0.0284	0.0240	0.0021	0.0144
standard error (SE)	0.0177	0.0156	0.0174	0.0135
p-value	p = 0.1585	p = 0.1754	p = 0.9090	p = 0.3275
95% confidence interval	−0.0148, 0.0716	−0.0142, 0.0622	−0.0406, 0.0447	−0.0187, 0.0475
**Population Mean Intercept** [ms: milliseconds]	21.3103	18.3875	21.4185	18.7656
standard error (SE)	0.8234	1.3216	0.6752	0.6972
p-value	p < 0.0001	p < 0.0001	p < 0.0001	p < 0.0001
95% confidence interval	19.2956, 23.3249	15.1536, 21.6213	19.7663, 23.0708	17.0597, 20.4715

Activating the isolated-pelvic coordination pattern, scaling PFM voluntary activation (columns 2 & 4) was not associated with induction of MEPs in the GMM, as expected from the EMG study results. Maximum co-activation of GMM [%-GMM-MVC] was small: 4.6501 while stimulating SMA and 3.9290 while stimulating M1-L.

The onset difference was significant, the onset of MEPs induced in the PFM happened after the onset of the MEPs induced in the GMM, regardless of the activation level of the GMM. While stimulating SMA, estimated intercept difference [onset of PFM MEP - onset of GMM MEP] = 2.7468 ms, SE = 0.7532, p = 0.0003, 95%CI (4.2259, 1.2678). While stimulating M1-L, estimated intercept difference [onset of PFM MEP - onset of GMM MEP] = 2.5997 ms, SE = 0.7994, p = 0.0012, 95%CI (4.1694, 1.0301).

Each PFM hotspot, SMA and M1-L, was stimulated under different levels of voluntary pre-activation.

Statistics are based on linear mixed effects regression models; see METHODS section.

PFM (pelvic floor muscles); GMM (gluteus maximus muscle).

Vol. PFM: Scaling PFM voluntary activation.

Vol. GMM: Scaling GMM voluntary activation.

Finally, during the activation of the gluteal-pelvic coordination pattern and regardless of activation level of the GMM, the onset of MEPs induced in the PFM was about 3 ms behind the onset of the MEPs induced in the GMM (Fig. 4C, Table 3C). The onset difference was significant: the onset of MEPs induced in the PFM happened after the onset of the MEPs induced in the GMM, regardless of the activation level of the GMM (Fig. 4C, Table 3C). Additionally, MEP onsets were not significantly modulated by voluntary drive: we found that increasing activation level of the GMM was not associated with change in the onset of MEPs induced in the GMM or in the PFM (Table 3C). We note that the intensity of the TMS stimulation we used did not produce supraphysiological EMG in the GMM: minimum MEP magnitudes were significantly less than maximum unperturbed background EMG activity in the 20 ms prior to the TMS pulse (p = 0.0141, paired t-test). Collectively, these results may indicate a lack of TMS evidence that there is a subcortical delay in excitation of gluteal activation relative to pelvic floor activation when activating the gluteal-pelvic coordination pattern.

To summarize, TMS results suggest that activating the isolated-pelvic coordination pattern modulates motor cortical excitability of pelvic-specific neurons in both SMA and M1, while activating the gluteal-pelvic coordination pattern modulates motor cortical excitability of the pelvic-specific neurons as well as the intermingled gluteal-specific neurons in both SMA and M1. Collectively, these results confirm that PFM representation is not just localized in SMA but extends to M1, and that the PFM and the synergistic GMM may be coordinated via the activation of intermingled cortical neurons in both SMA and M1.

## Discussion

**Figure 5:**
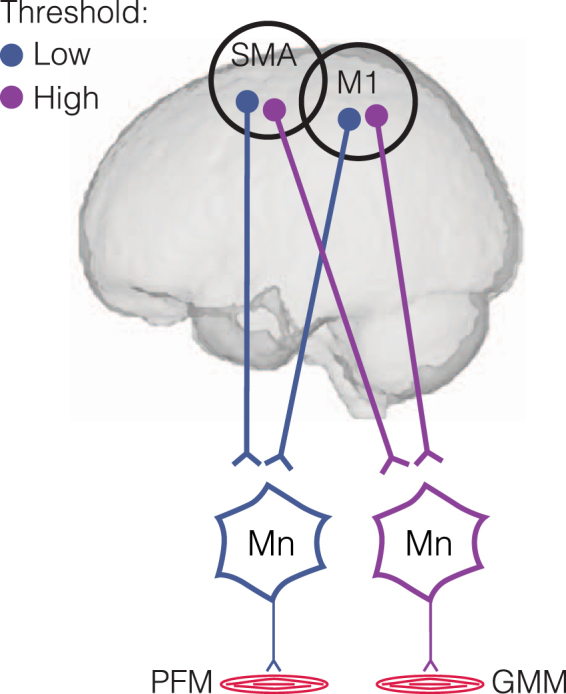
Schematic representation of proposed cortical representation of the human pelvic floor. We proposed that, similar to other muscles such as the gluteus maximus muscle (GMM), pelvic floor muscles (PFM) have a distributed representation in the supplementary motor area (SMA) and the primary motor cortex (M1). We further propose that the PFM representation has a lower activation threshold than synergistic muscles such as the GMM. This activation threshold difference may explain why the PFM is activated in advance of the synergists during cortically-driven activation. Mn = Motor neuron.

Here we find that the pelvic floor muscles (PFM) are represented broadly in the human motor cortex, both in SMA and M1. Our results suggest that the cortical representation controlling PFM overlaps with the cortical representation controlling synergistic muscles (such as the gluteals, GMM); however, neurons comprising the pelvic floor representation may be distinct from the synergist representation in activation threshold. Furthermore, our results suggest that the PFM motor cortical representation may be accessed independently of the synergist representation, yet PFM and their synergistic muscles may be cortically coordinated via the co-activation of intermingled neurons. We graphically summarize our findings (Fig. 5).

Traditionally, the motor cortical representation of the PFM has been thought to be dominated by a region in the medial wall of the precentral gyrus toward SMA^1,2,11,12,29–32^, and the contribution of M1 has not been fully recognized^11,29–32^. There are at least three reasons why the M1 representation of the PFM has not been apparent in previous studies. First, studies that use only fMRI and seek specificity in the pelvic floor representation by contrasting brain activity during pelvic floor contraction with brain activity during contraction of another muscle may not have adequately considered co-activation patterns of the two muscles. For example, pelvic floor contraction has been contrasted with toe muscle contraction^12^, but we have recently shown that pelvic floor and toe muscles co-activate during muscle contraction^11^. Therefore, assuming that M1 representations of the pelvic floor and the synergist overlap, the contrast may downplay the contribution of M1. Second, previous studies did not typically examine the PFM with TMS in addition to fMRI as we have done here; in a single study that did use TMS, separate M1 and SMA activation hotspots for the PFM appeared to emerge^27^. Third, and perhaps most interestingly, the choice of the experimental excitation of the pelvic floor could engage different brain networks that are differentially connected to SMA versus M1. We have recently confirmed in humans that trunk muscles have both an SMA and M1 representation, but using resting state functional connectivity, we found that these representations appear to be communicating with different brain networks^26^. We have previously published preliminary evidence that this dual representation also applies to the PFM, with the SMA component of the representation preferentially communicating with brain areas known to be involved in the sensory representation of bladder state (such as the posterior insula)^11^. Therefore, studies that excite the pelvic floor via bladder filling may preferentially activate SMA compared to M1^29,30,32^. Our present study suggests that the M1 representation of the PFM is at least as strong as the SMA representation of the PFM, and enables new studies to decode how these regions may cooperate to achieve different types of tasks.

Our results, also, relate to a long-standing debate about the source of neural coupling that assembles muscles into functional groups, termed *muscle synergies*. Work in animal and human models suggest that circuits of sub-cortical interneurons may encode specific patterns of muscle activity that can be activated by cortical neurons^33–38^. Additionally, a different form of muscle coordination is that coordination of muscles can be structured at a cortical level^22–24,39–44^. In this view, motor cortical output can independently reach different muscles, but is automatically coordinated at the cortical level during task performance^45^. Our results are in line with previous suggestions of cortical structuring of muscle synergies by overlapping motor representations^23–25^. However, our work goes on to make the novel suggestion that temporal characteristics of muscle coordination may be encoded by differences in excitability of separate motor cortical populations. Our TMS results provide evidence that the two intermingled populations that control the PFM and GMM are distinct in their excitability thresholds – relatively low threshold for the PFM and relatively high threshold for the GMM. If a common input is distributed to both populations during voluntary activation of the gluteal-pelvic coordination pattern, recruitment of the motor cortical neurons for the PFM would potentially occur first, analogous to the size principle of spinal motor neuron recruitment^46^. Lower excitability threshold of the motor cortical neurons that control the PFM in SMA and M1 could be the result of differences in motor cortical neuron size for different muscles^47^, or the result of GMM-independent and PFM-specific excitatory pre-synaptic inputs to continuously regulate urine storage^1,2,6,48,49^.

Our work is limited in that we did not study other movements that co-activate the PFM to test generalizability of our findings; for example, finding representations of the PFM intermingled with other coordinated trunk and arm muscles (e.g. deltoid, see^50^). Also, it is possible that the representation of the PFM could be localized to SMA, and apparent activation of the PFM during M1 stimulation may results from stimulus spread. We do not find this explanation plausible for three reasons. First, this would not explain the scaling of BOLD signal in M1 during activation of the isolated-pelvic coordination pattern when the GMM muscle is inactive and activity in other non-PFM muscles (such as the abdominals) is likely quite modest during submaximal PFM contraction^51^. Second, responses in the PFM to stimulation of M1 were no smaller than responses to stimulation of SMA, which would be expected as the stimulation moved farther away from the true representation. Third, data in previous TMS studies suggest separate representations of the PFM in SMA and M1^27^.

Our work has implications for localizing dysfunction in disorders of the human pelvic floor. In previous studies, we have focused on the medial wall of the motor cortex, in particular SMA, as the main location of PFM motor cortical control, as well as a possible contributor to urologic chronic pelvic pain syndrome (UCPPS)^9,11,13,14^. Our recent resting state fMRI research provides evidence of altered motor control in patients with UCPPS, based on SMA frequency content and SMA connectivity with other brain regions^13,14^. Furthermore, our novel findings extend the representation and control of the PFM to motor regions beyond the medial wall of the motor cortex; thus, expanding our discovery process to new areas that were not previously considered in research on UCPPS. Therefore, basing our analysis on PFM-specific M1, may provide additional evidence related to the contribution of M1 to UCPPS and/or the distinction between the representation of the PFM in SMA and M1. These findings may be useful in understanding underlying mechanisms visceral sensitivity in different disorders that are associated with chronic pelvic pain; for example, comparing patients with UCPPS to patients with functional gastrointestinal disorders. Furthermore, these findings may be useful to extend promising research on the analgesic effects of repetitive TMS in UCPPS^17,52,53^, and anorectal disorders^8^, by providing additional potential targets (either active or control).

## Methods

### Participant population

We performed three studies and recruited a total of 43 healthy participants with age (mean ± SD) of 30.0 ± 5.5 (range: 22–46 years). Participants included 25 women with age 29.2 ± 6.1 (range: 22–46 years) and 18 men with age 28.7 ± 4.6 (range: 24–43 years). There was no difference in age by sex (p = 0.7628, unpaired t-test). Participants could be included if they were older than 18 years of age, were able to participate in the informed consent process, were safe to be scanned by magnetic resonance imaging, were safe to be stimulated with transcranial magnetic stimulation, and reported no history of neurological conditions or chronic pain. Female participants were deferred if they were in the menstrual phase. In each study, participants were asked to void at the beginning of the experimental session so that they would have no bladder or bowel urgency during the session – no sessions were interrupted due to urgency. Each study was completed in a single day with each participant. We performed these studies at the University of Southern California (USC), and the USC Institutional Review Board approved all procedures. All aspects of the study conformed to the principles described in the Declaration of Helsinki. All participants provided informed consent.

### Overview of methods

In order to determine whether the pelvic floor motor cortical representation is localized or distributed we performed three separate studies using different experimental modalities: electromyography (EMG), functional magnetic resonance imaging (fMRI), and transcranial magnetic stimulation (TMS). All studies focused on two main types of coordination patterns that involve the pelvic floor muscles (PFM): “isolated-pelvic” pattern that activates the PFM independently of the gluteus maximus muscle (GMM, a non-PFM synergistic muscle group; PFM is the primary muscle) and “gluteal-pelvic” pattern that co-activates the PFM with the GMM (GMM is the primary muscle). These coordination patterns have been described previously^9,50,54^, but have not been fully characterized at either the muscle or brain level. The isolated-pelvic coordination pattern is elicited by instructing participants to contract their pelvic floor as if to stop the flow of urine (note that it does not involve an instruction to “isolate” the PFM). The gluteal-pelvic coordination pattern is elicited by instructing participants to contract their gluteal muscles (note that it does not involve an instruction to co-activate the PFM). We hypothesized that motor cortex contains a location that primarily represents the PFM in the medial wall of the precentral gyrus located anteriorly toward the supplementary motor area (SMA)^9,10,12,27^ and a bilateral representation specific to the GMM located posteriorly toward the primary motor cortex (M1). We hypothesized that the isolated-pelvic coordination pattern is produced by activity focused in the localized PFM representation in SMA, and that the gluteal-pelvic coordination pattern is structured in the cortex by co-activation of the separate and localized PFM and GMM representations. In the first study, we used EMG to determine if isolated-pelvic and gluteal-pelvic coordination patterns were stable; i.e., they would be observed at different levels of voluntary drive. In the second study, we tested our hypotheses by first localizing the hypothesized PFM and GMM representations, and then examining how brain activity would change in these regions as the voluntary drive to elicit isolated-pelvic, or gluteal-pelvic, coordination pattern was varied. In the third study, we tested the specificity of the hypothesized PFM and GMM representational areas by determining how their motor cortical excitability would vary as the voluntary drive to elicit isolated-pelvic, or gluteal-pelvic, coordination pattern was varied. The following sections describe the acquisition and analysis methods for each modality (EMG, fMRI, and TMS).

#### EMG acquisition

In forty-three healthy participants (25 women + 18 men), we measured muscle activation using EMG to define characteristics of PFM activation patterns (isolated-pelvic and gluteal-pelvic); we did not analyse EMG data from one female participant because it was an incomplete dataset. We also analysed activation of a finger muscle to establish an appropriate control muscle group that is not associated with coordinated PFM activity^9^. We chose the right first dorsal interosseous muscle (FDI) as the control muscle because we have previously verified that the FDI does not co-activate with the PFM^9^. We did not choose a trunk or a lower limb muscle as the control because of the known synergistic coupling between PFM and surprisingly distal muscles, such as the toe and shoulder muscles^11,50^.

With the participant resting in a supine position, we recorded surface EMG data from the PFM, the right GMM, and the right (FDI). We recorded an EMG signal from the PFM using a medical-grade rectal EMG sensor (Pathway Rectal EMG Sensor, The Prometheus Group, Dover, NH), which provided a bipolar recording from two bar electrodes – 12 mm apart, with dimensions 30 mm by 7 mm – mounted longitudinally along a cylindrical plug-type applicator. The rectal EMG sensor is 50 mm long with a stopper that ensures that one end of the sensor rests stably at the anal verge. The rectal EMG sensor likely recorded an aggregate signal from the PFM that included the anal sphincter and levator ani, with possible small contributions from more distant muscles^55^. Activity of the PFM has been previously measured using a rectal sensor, and does not contain significant cross-talk from the GMM^9,50,54^. We recorded EMG signals from the GMM and FDI with miniature electrode/preamplifiers (DELSYS, Boston, MA) with two silver recording surfaces, 5 mm long and 10 mm apart. The EMG preamplifier filters had a bandwidth of 20–450 Hz, with gains of 10,000 for the PFM and 1000 for the GMM and FDI, and a sampling rate of 2000 Hz.

Participants performed separate trials, each of which involved voluntary activation of a different coordination pattern. For all trial types, we first acquired EMG data corresponding to maximal voluntary contraction (MVC) of the given coordination pattern, by adopting the same conditions of the following activation trials. In finger trials, we instructed participants to contract their FDI muscle to generate index finger abduction. In isolated-pelvic trials, we instructed participants to contract their PFM as if to stop the flow of urine. In gluteal-pelvic trials, we instructed participants to “squeeze” their GMM. For each coordination pattern, participants received visual EMG feedback from the primary muscle guiding them to produce muscle contractions to 15%, 35%, and 45% MVC (in random order across participants). For each coordination pattern, each participant produced 20 contractions per %MVC level. Each contraction was cued randomly in time. Once a given contraction was cued, an audio signal ramped up and down in pitch to guide the participant through a smooth activation/deactivation over a period of 2 seconds.

#### EMG analysis

We processed the EMG data to calculate (1) the onset of each voluntary activation for each primary muscle group, (2) the onset of associated activations for the non-primary muscle groups, and (3) the maximum amplitude of the activity profile for each muscle. EMG signals from all recorded muscles were first high-pass filtered at 100 Hz (fourth-order zero-lag Butterworth filter), rectified, low-pass filtered at 30 Hz^9,50^, and then normalized to identically processed EMG data from the maximum activation trial. EMG data were then smoothed with a 500 ms moving average. Activation onsets were defined to occur when the smoothed EMG exceeded 2 standard deviations (SD) of the EMG baseline noise with the muscle at rest. We have used these methods in our prior work^9,11^; however, in the present work we did not average EMG signals across repeated activations in each participant (Fig. 1B). Each activation was quantified and expressed as percentage MVC (expressed as %MVC). All participants performed repeated and scaled muscle contractions; therefore, each participant provided multiple EMG data points for each muscle group that were used in the group analyses.

To evaluate the statistical significance of changes in magnitude and timing of muscle activation associated with increases in voluntary activation of the primary muscle of the task (Fig. 1C), we used linear mixed effects regression models with participant-level random intercepts and random slopes (SAS version 9.4, SAS Institute). First, we report the statistical significance of the regression slopes relating changes in muscle activation magnitude to changes in the activation magnitude of the primary muscle – i.e., βo in *Y*_ij_ = αo + (βo + bo_i_) * *X*_ij_ + uo_i_ + ε_ij_, where *Y*_ij_ was defined as the EMG co-activation in the muscle of interest (non-primary muscle) and *X*_ij_ was defined as the EMG activation in the primary muscle, for the j^th^ activation by the i^th^ participant. Additionally, we report the statistical significance of the difference in regression slopes, based on the primary muscle group, relating changes in muscle activation magnitude to changes in primary muscle activation magnitude – i.e., β1 in *Y*_ij_ = (αo + uo_i_ + (α1 * *X2*_i_)) + (βo + bo_i_ + (β1 * *X2*_i_)) * *X1*_ij_ + ε_ij_, where *Y*_ij_ was defined as the EMG co-activation in the muscle of interest, *X1*_ij_ was defined as the EMG activation in the primary muscle, *X2*_i_ was defined as the primary muscle identifier (one of two primary muscles), and (*X1*_ij_ * *X2*_i_) was the interaction term of interest, for the j^th^ activation by the i^th^ participant. Finally, we report the statistical significance of the regression slope relating changes in muscle activation latency to changes in primary muscle activation magnitude – i.e., βo in *Y*_ij_ = αo + (βo + bo_i_) * *X*_ij_ + uo_i_ + ε_ij_, where *Y*_ij_ was defined as the onset difference between the muscle of interest and the primary muscle (ms) and *X*_ij_ was defined the EMG activation in the primary muscle, for the j^th^ activation by the i^th^ participant.

#### fMRI acquisition

We measured brain activation associated with the voluntary muscle activation tasks (described above) using fMRI. To ensure the repeatability of our findings, we used two separate participant groups (A and B). In a first group of healthy participants (Group A: n = 23, 14 women + 9 men; Fig. 2A), we used fMRI recordings during blocks of comfortable GMM and FDI voluntary contraction to localize motor regions of interest (ROIs) related to activation of the gluteal-pelvic coordination pattern, since the gluteal-pelvic coordination pattern involves both the PFM and GMM. In another group of healthy participants (Group B: n = 20, 11 women + 9 men; Fig. 2B), we used fMRI recordings during blocks of scaled muscle contraction (three distinct levels) to examine changes in brain activity (as measured by BOLD signal strength) in the identified motor ROIs during voluntary activation of the isolated-pelvic or gluteal-pelvic coordination patterns. We used a 3-tesla scanner (GE Signa Excite) with an eight-channel head coil. We positioned participants supine while viewing a fixation crosshair, and placed foam pads to limit head motion. As in our previous fMRI studies of PFM activation^9,11^, we collected T2-weighted echo planar image volumes with blood oxygen level-dependent (BOLD) contrast (echo time, 34.5 ms; flip angle, 90°; field of view, 220 mm; pixel size, 3.43 mm) continually every 2.5 s during two imaging runs. Each volume consisted of 37 axial slices (3 mm slice thickness, 0.5 mm interslice gaps) that covered the brain from vertex to cerebellum. We additionally acquired a T1-weighted high-resolution anatomical image from each participant, for the purpose of spatially registering functional images. For Group A (Fig. 2A), we cued participants to voluntarily activate each muscle group (to ~20% effort) in two separate runs, FDI activation run followed by GMM activation run. Participants performed 6 activation blocks in the scanner, during each block they contracted and relaxed every 2 seconds following a flashing crosshair. For Group B (Fig. 2B), we cued participants to voluntarily activate each muscle group (to three distinct levels: low (L), medium (M), high (H)) in two separate runs, PFM activation run followed by GMM activation run. Participants performed 6 activation blocks in the scanner (L, M, H, L, M, H), during each they contracted and relaxed following a flashing crosshair. The EMG sensors were not used during the fMRI acquisition. Therefore, before entering the actual MRI scanner, all participants were trained on producing muscle activation patterns in the FDI, PFM, and GMM similar to the activation patterns observed during the EMG study, while receiving the same instructions on how to contract each muscle group. This training was performed in a mock MRI scanner to ensure an identical environment and experimenters verified the EMG activation patterns during the training step. Participants in Group A were trained to produce one low level of muscle activation, while participants in Group B were trained to produce distinct L, M, and H activation of the isolated-pelvic and gluteal-pelvic coordination patterns.

#### fMRI preprocessing for both groups

For participants in both groups, we preprocessed each participant’s fMRI data using the FMRIB Expert Analysis Tool (FEAT, http://www.fmrib.ox.ac.uk)^56^, which included skull extraction using the brain extraction tool (BET) in FSL (FMRIB Software Library), slice timing correction, motion correction, and spatial smoothing using a Gaussian kernel with full-width half-maximum of 5 mm and nonlinear high-pass temporal filtering (100 s). We used a general linear model (GLM) to examine the changes in BOLD signal associated with muscle activation for the three tasks (FDI and GMM in Group A, PFM and GMM in Group B). We performed participant-level whole-brain GLM analyses of individual runs in each participant to determine the change in BOLD signal during the activation blocks compared with the rest blocks.

### fMRI processing for Group A

We then performed a group-level mixed-effect (FLAME 1 in FSL) analysis, with unpaired two-sided t-tests of voxel-wise regression coefficients, to identify voxels in standard Montreal Neurological Institute (MNI) coordinates with significant differences in response based on the muscle group being voluntarily contracted by the participant. The contrast of interest was GMM > FDI; i.e., (GMM > Rest) > (FDI > Rest). We thresholded the group-level image for this contrast with cluster-based correction for multiple comparisons (Z > 2.3 and p < 0.05). Finally, using the thresholded group-level image and the Jülich Histological Atlas within FSL^57^, we calculated motor cortical ROIs for specific Brodmann areas (BA: BA6 for supplementary motor cortex [SMA], BA4 for bilateral primary motor cortex [M1-L, M1-R]; Fig. 2A). Each ROI only included voxels that were statistically significant in the GMM > FDI contrast and had ≥25% probability of belonging to the BA of interest.

### fMRI processing for Group B

We then projected each motor cortical ROI (SMA, M1-L, M1-R, identified by processing Group A data at the group level) into the native space of each participant in Group B to select voxels that were active (Z > 2.3, p < 0.05, cluster corrected) in the contrast of muscle activity compared to rest – we tested both GMM > Rest and PFM > Rest – regardless of the contraction level. For each participant, contracting muscle, and ROI, BOLD signals observed from the selected voxels were averaged; therefore, each participant provided six averaged BOLD signals (3 ROIs x 2 activation runs [PFM, GMM]; Fig. 2B). Finally, to evaluate the statistical significance of changes in the averaged BOLD signal in association with increases in voluntary activation of each primary muscle group, we used analysis of covariance (ANCOVA, SAS version 9.4, SAS Institute). ANCOVA was used to evaluate whether population means of the dependent variable (BOLD signal strength during muscle activation blocks) are equal across levels of the categorical independent variable (*activation level*), while statistically controlling for the effects of another variable that is not of primary interest (*BOLD signal strength during rest blocks*). To perform the analysis, we calculated a representative value of BOLD signal strength during an activation block (*A*) and a representative value of BOLD signal strength during the preceding rest block (*R*), for each activation level. *A* was defined as either the 50^th^ or 75^th^ percentile of a BOLD signal interval that corresponded to an activation block, while *R* was defined as the 50^th^ percentile of the preceding BOLD signal interval that corresponded to the preceding rest block. Results for defining *A* were not affected by choosing the 50^th^ or 75^th^ percentile, and only the results from the 75^th^ percentile are reported for simplicity. An ANCOVA model (*A*_ij_ = Intercept + β_1_ * *L* + β_2_ * *M* + (0) * *H* + β_3_ * *R*_ij_ + *U*_i_ + *E*_ij;_ i was participant identifier [1 to 20] and j was block identifier [1 to 6]) was fit to all data from all 20 participants in Group B, per contracting muscle (PFM, GMM) and per ROI (SMA, M1-L, M1-R). The ANCOVA model assumed that each participant had a unique BOLD baseline (Intercept + Ui)^58,59^. BOLD variability during an activation block can be affected by BOLD variability during the preceding rest block due to brain-physiology-related low-frequency drifts in BOLD^60^; therefore, we used ANCOVA analysis to include rest blocks as baseline measurements of the outcome variable that is BOLD signal strength during muscle activation blocks. *L*, *M*, and *H* were the identifiers of low, medium, and high activation levels, respectively. Participants repeated each activation level twice; therefore, the extracted BOLD signal could be divided into 6 active intervals/blocks interleaved with 6 preceding rest intervals/blocks. For each contracting muscle and ROI, we report the statistical significance of changes in BOLD signal strength when increasing muscle activation magnitude, by comparing BOLD signal strength during low-level activation to BOLD signal strength during high-level activation (β_1_ slope) and by comparing BOLD signal strength during medium-level activation to BOLD signal strength during high-level activation (β_2_ slope).

#### TMS acquisition

In seven healthy participants (4 women + 3 men, a convenience sample from Group B in the fMRI data collection), we obtained motor evoked potentials (MEPs) from the PFM and right GMM, with participants resting supine, while using a single-pulse biphasic magnetic stimulator (Magstim Rapid^2^, Magstim) and a 110-mm double-cone coil (Magstim). The stimulation system was integrated with the EMG system of the Brainsight Frameless System (Rogue Research). Brainsight EMG system had an overall EMG amplification of 2500, an overall bandwidth of 16–470 Hz, and a sampling rate of 3000 Hz per channel. We recorded EMG signals from the PFM and GMM, using electrodes similar to the electrodes used in the EMG study described above. After acquiring a few visible MEPs, Brainsight *MEP-selection window* was adjusted and fixed throughout the entire TMS session to avoid including stimulation artefacts in calculating MEP peak-to-peak amplitude and onset. The MEP-selection window was 47 ms on average, extending from 13–60 ms after each TMS pulse.

fMRI results (Fig. 2B) did not clearly support the hypothesis of localized motor cortical representation of the PFM, and led us to hypothesize that the motor cortical representation of the PFM is not only localized to the medial wall of the precentral gyrus toward SMA and can be intermingled with the motor cortical representation of their synergists, i.e., the GMM. Therefore, the TMS procedure involved the following steps that were applied to each participant: (1) Mapping the medial motor cortex to identify PFM hotspot(s); (2) Calculating PFM resting motor threshold (rMT) to use as the TMS intensity during muscle contraction; and, (3) Calculating peak-to-peak amplitude and latency onset of MEPs induced during muscle contraction. After mapping the medial motor cortex, we focused on stimulating the identified hotspot in SMA and in M1 in the left hemisphere (corresponding to M1-L in fMRI), without stimulating the identified hotspot in M1 in the right hemisphere (corresponding to M1-R in fMRI), to reduce data collection time and stimulation risks.

### PFM stimulation at rest for mapping, hotspots, and motor threshold

We constructed a grid of stimulation points with spatial resolution of 7 × 7 mm using Brainsight Frameless System (Rogue Research). In each participant, the stimulation grid covered the medial motor cortex, including SMA and bilateral M1 (extending **~**21 mm lateral to the midline). We verified that the grid covered all activated motor regions during GMM voluntary activation by overlaying the grid on top of the first-level fMRI statistical image obtained by analysing the participant’s fMRI data from the GMM activation run (contrast: GMM > Rest). Prior to stimulation, we pre-selected a hypothetical PFM hotspot along the medial wall of the motor cortex, guided by the participant’s own fMRI GMM activation map. We then stimulated this hypothetical hotspot to select a stimulation intensity to use during the mapping procedure. We selected the smallest stimulation intensity that achieved 100% success rate and highest consistency of MEPs induced in the PFM. Stimulating at the selected intensity, each grid point was stimulated three times with a 6-second interstimulus interval (ISI). Grid points were stimulated in a randomized order selected by Brainsight (Brainsight 2.3.3). Combining reasonably short ISI and random walk between grid points has been validated to produce reliable brain maps, while lowering mapping time and number of stimuli without increasing chances of TMS-induced plasticity^61^. Hotspots were then selected for SMA and M1, using a motor mapping algorithm offered by Brainsight software and by inspecting the amplitude and consistency of the MEPs associated with prospective hotspots, as well as the underlying anatomical structures to identify SMA and M1. To visualize the group average motor map for the PFM (Fig. 3C), we first interpolated individual participants PFM maps into a 1-mm standard grid using natural neighbour interpolation (*griddata* in MATLAB), normalized these maps to their maximum MEP value, and finally averaged the standardized and normalized maps.

Muscle contraction is known to lower motor cortical excitability^62–64^; therefore, we aimed to stimulate identified PFM hotspots at their rMT – as a conservatively low stimulation intensity – while testing our hypotheses. At each PFM hotspot, we began by stimulating the hotspot at 4–5 intensities (above and under the mapping intensity, 5 stimuli per intensity at 6-second ISI). PFM rMT was selected as the percentage of maximum stimulator output (%MSO) with ≈ 50% success rate of eliciting consistent MEPs in the PFM. We then stimulated identified hotspots at rMT during muscle activation, as described in the next section.

### PFM-hotspot stimulation during muscle activation

Participants performed separate trials (Fig. 4B), each of which involved voluntary pre-activation of a different primary muscle group (PFM followed by GMM) and single pulse TMS. For all trial types, we first acquired EMG data corresponding to MVC. For each primary muscle group, participants received visual feedback guiding them to sustain muscle contractions to 20%, 35%, and 50% MVC, while individual PFM hotspots were stimulated at the identified rMT. For each voluntarily contracting muscle, each participant produced 20 contractions per %MVC level with the potential of eliciting 40 MEPs due to simultaneous EMG data collection from the PFM and GMM.

#### TMS analysis

We processed the EMG data and TMS trigger signal to calculate the amplitude of the activity for each muscle at the time of applying TMS. Furthermore, we processed Brainsight EMG data to calculate peak-to-peak amplitude and onset of elicited MEPs. For each contraction, we collected from each Brainsight EMG channel (one for the PFM and one for the right GMM) a signal that extended for 150 ms, 50 ms before and 100 ms after the onset of the TMS pulse. Within each participant’s MEP-selection window, the onset of an MEP was assumed when the rectified EMG signal exceeded 2 standard deviations of a baseline signal that spanned the 20 ms leading to the onset of the TMS pulse. For each TMS pulse, Brainsight software (Brainsight 2.3.3) outputted the peak-to-peak absolute amplitude (in microvolts) of the recorded EMG signal within the MEP-selection window. For across-participant comparisons and group analyses, all MEP amplitude values were normalized. For each hotspot: PFM MEP amplitude values were normalized to the highest PFM MEP amplitude observed during both the PFM and GMM voluntary pre-activation trials. GMM MEP amplitude values were normalized to the highest GMM MEP amplitude observed during both the PFM and GMM voluntary pre-activation trials.

We have hypothesized changes in motor cortical excitability of pelvic- and gluteal-specific neural populations during the activation of the isolated-pelvic and gluteal-pelvic coordination patterns. Changes in motor cortical excitability can be quantified by assessing changes in MEPs’ peak-to-peak and onset values, while stimulating at a fixed intensity^28,62–64^. To evaluate the statistical significance of changes in amplitude (Fig. 4B) and timing (Fig. 4C) of MEPs associated with increases in voluntary pre-activation, we used linear mixed effects regression models with participant-level random intercepts and random slopes (SAS version 9.4, SAS Institute). First, we report the statistical significance of the regression slopes relating changes in amplitudes of MEPs (PFM and GMM) to changes in GMM pre-activation magnitude and to changes in PFM pre-activation magnitude – i.e., βo in *Y*_ij_ = αo + (βo + bo_i_) * *X*_ij_ + uo_i_ + ε_ij_, where *Y*_ij_ was defined as the amplitude of MEP induced in one muscle (PFM or GMM) and *X*_ij_ was defined the EMG pre-activation level in the same muscle at the time of the TMS pulse, for the j^th^ activation by the i^th^ participant. Finally, we report the statistical significance of the regression slope relating changes in MEP onset latency (onset of PFM relative to onset of GMM) to changes in GMM pre-activation magnitude, as well as the statistical significance of the difference in the intercepts – i.e., β1 and α1, respectively, in *Y*_ij_ = (αo + uo_i_ + (α1 * *X2*_i_)) + (βo + bo_i_ + (β1 * *X2*_i_)) * *X1*_ij_ + ε_ij_, where *Y*_ij_ was defined as the MEP onset (ms), *X1*_ij_ was defined as the EMG pre-activation level in the GMM at the time of the TMS pulse, *X2*_i_ was defined as the MEP identifier (MEP observed either in PFM or in GMM), and (*X1*_ij_ * *X2*_i_) was the interaction term of interest regarding difference in the slopes, for the j^th^ GMM activation by the i^th^ participant.

### Data availability

The datasets generated during and/or analysed during the current study are available from the corresponding author on reasonable request.
